# Impact of Topical Fluocinonide on Oral Lichen Planus Evolution: Randomized Controlled Clinical Trial

**DOI:** 10.1111/odi.15156

**Published:** 2024-10-14

**Authors:** Alessandro Polizzi, Gianluca Martino Tartaglia, Simona Santonocito, Angela Alibrandi, Anna Elisa Verzì, Gaetano Isola

**Affiliations:** ^1^ Department of General Surgery and Surgical‐Medical Specialties, School of Dentistry University of Catania Catania Italy; ^2^ Department of Biomedical, Surgical and Dental Sciences University of Milan Milan Italy; ^3^ Fondazione Ca'Granda IRCCS Ospedale Maggiore Policlinico Milan Italy; ^4^ Department of Economics, Unit of Statistical and Mathematical Sciences University of Messina Messina Italy; ^5^ Department of General Surgery and Surgical‐Medical Specialties, Unit of Dermatology University of Catania Catania Italy

**Keywords:** anxiety, clinical study, depression, fluocinonide, oral lichen planus, oral mucosa, randomized clinical trial, stress, treatment

## Abstract

**Objective:**

To examine the impact of fluocinonide 0.05% gel formulation for the topical treatment of oral lichen planus (OLP).

**Methods:**

Through an RCT design, 47 patients with OLP were randomly allocated for topical OLP treatment with fluocinonide 0.05% (*n* = 23) or placebo (*n* = 24). Patients were examined for OLP symptoms, signs, disease severity, and extension score changes over 6‐month follow‐up.

**Results:**

After 6 months, in comparison with placebo, patients treated with fluocinonide experienced a significant reduction of OLP symptoms (*p* = 0.024), signs (*p* = 0.014), and OLP extension score (*p* = 0.028). The two‐way ANOVA estimation models revealed that treatment with fluocinonide determined, at 6 months, a positive significant effect on the reduced OLP signs (*p* = 0.017), OLP symptoms (*p* = 0.026), and OLP extension score (*p* = 0.028). The multivariate regression analysis highlighted that anxiety, stress, and depression were significant predictors of every analyzed OLP outcome (*p* < 0.05 for each parameter) and that patients who had baseline anxiety, depression, and stress gained more benefits from fluocinonide at 6‐month follow‐up.

**Conclusions:**

Topical fluocinonide 0.05% was more efficacious compared to placebo in reducing OLP outcomes at 6‐month follow‐up. Anxiety, depression, and stress were significant predictors of OLP outcomes and positively impacted the treatment with fluocinonide at 6 months.

## 1 Introduction

Oral lichen planus (OLP) is a chronic inflammatory mucocutaneous disorder affecting the oral mucosa, with an estimated prevalence ranging from 0.5% to 2.2% of the global population (Gorouhi, Davari, and Fazel 2014; Warnakulasuriya et al. 2021). Despite its occurrence, the etiology of OLP remains still unclear; it has been reported that its pathogenesis involves immune‐mediated mechanisms through the autoimmune apoptosis by activated cytotoxic CD8+ T‐lymphocytes and microRNA dysregulations (Polizzi et al. 2024) that, together with both exogenous and endogenous factors, could influence OLP clinical appearance and evolution (Isola et al. 2023; Lavanya et al. 2011; Liu et al. 2023; Zhou et al. 2002). A wide range of clinical manifestations of OLP have been reported, which encompass a spectrum of lesions from reticular patterns to erosive and ulcerative forms, leading to substantial morbidity and could impact the overall quality of life in OLP patients (Arduino et al. 2023; Carrozzo et al. 2019). It has recently been shown that the chronic inflammatory stimulus associated with OLP could determine, if not diagnosed and treated promptly, primary subclinical evidence of the potentially malignant transformation of OLP (Luis et al. 2022). In this regard, while the average risk of evolution of OLP in oral squamous cell carcinoma (OSCC) is around 1.37% (Warnakulasuriya and Lodi 2021), several local chronic environmental and inflammatory factors have been reported to significantly increase the risk of OLP degeneration (González‐Moles et al. 2019).

During the last few decades, a wide range of topical pharmacological strategies have been reported to prevent OLP malignant progression. Between those, tacrolimus (Polizzi et al. 2023) and glucocorticoids such as clobetasol have been evidenced as valuable therapies in the early management of OLP through their impact on the inflammatory T cell‐mediated, strictly linked with OLP evolution (Kerr and Lodi 2021; Lodi et al. 2020; Santonocito et al. 2021). Among glucocorticoids, fluocinonide has been demonstrated, in some systemic disease models, due to its concomitant anti‐inflammatory and immunosuppressive properties, to be promising in the treatment of some similar OLP inflammatory cutaneous conditions such as dermatitis (Del Rosso and Bhambri 2009), psoriasis (Lebwohl et al. 1998) and mucosal lichen planus (Davari, Hsiao, and Fazel 2014). More specifically, in topical formulation, a preclinical trial reported that fluocinonide, in a protocol that included chlorhexidine and miconazole, was able to both significantly reduce certain clinical OLP symptoms (Carbone et al. 1996) and, at the same time, to be valuable in the cost‐effective OLP management through its reduced side effects (Sandhu et al. 2022).

Based on this evidence, the objectives of the present study were to evaluate the effectiveness of the oral, topical formulation of fluocinonide at 6‐month follow‐ups in patients with OLP by comprehensively analyzing the effects of fluocinonide on clinical features such as OLP signs and symptoms and severity at 6‐month follow‐up. Moreover, it assessed possible clinical predictors linked with OLP evolution and whether patient‐related baseline OLP variables impacted the effectiveness of OLP treatment protocols at 6‐month follow‐up.

## 2 Materials and Methods

### 2.1 Design of the Study

The present study was designed as a triple‐blinded, randomized‐controlled clinical trial (RCT) aimed to evaluate a topical formulation of fluocinonide compared with a placebo at 6‐month follow‐up in patients with OLP. For the study, patients with OLP were recruited at the Dental School of the University of Catania, Catania, Italy, from September 2023 to February 2024. The study performed the guidelines of the Helsinki Declaration on medical research reviewed in 2013 and the CONSORT guidelines (Moher et al. 2012). The study protocol (prot. 7/24/23‐PAR) was approved by the International Review Board at the University of Catania, Catania, Italy, and registered on clinicaltrials.gov (NCT 06135805). All patients were informed about the study characteristics and risks and signed their consent before their enrollment.

### 2.2 Study Population

OLP patients who adhered to the World Health Organization (WHO) 2020 clinical and histopathological OLP criteria (Warnakulasuriya et al. 2021) were enrolled in the present study. Accordingly, the inclusion criteria were (1) < 18 years old; (2) presence of symptomatic OLP lesions; (3) a network of white lines arranged in a reticular, annular, or linear pattern that resembles lace, either with or without erosions and ulcerations; (4) the potential for OLP‐related desquamative gingivitis intended as erythema, desquamation, erosion, and eventual blistering of the attached and marginal gingiva (Prinz 1932); (5) a distinct zone of cellular infiltration, resembling a band, restricted to the superficial lamina propria and predominantly composed of lymphocytes; (6) vacuolar degeneration and keratinocyte apoptosis in the basal/suprabasal cell layer; (7) possibility of atrophic variety with epithelium thinning and sometimes ulceration and mixed inflammatory infiltration. Patients with erosive OLP lesions without symptoms were also included.

The exclusion criteria were: (1) any OLP treatment administered in the 6 months before the study; (2) white OLP lesions without symptoms (Brennan et al. 2022); (3) status of pregnancy or lactation; (4) contact lichenoid lesions or oral drug‐related lesions; (5) allergy or intolerance to drugs; (6) heavy drinking history.

An amnestic questionnaire was used to assess each patient's medical history and the presence of comorbidities. Anxiety, depression, and stress were assessed using the combined Depression Anxiety Stress Scale‐21 (DASS‐21) (Lovibond and Lovibond 1995; Manczyk et al. 2019), consisting of 21 items equally divided to evaluate depression, anxiety, and stress separately (seven questions grouped per single domain). Patients were asked to assess their symptom experience using a four‐point scale, ranging from 0 (“did not apply to me at all”) to 3 (“applied to me very much or most of the time”) for each item. Depending on the al score resulting from the sum of the items, the severity of a specific negative emotion was categorized as normal, mild, moderate, severe, or extremely severe. Data were recorded at baseline and at 6 months of treatment.

Patients were categorized based on their smoking history as never‐smokers, ex‐smokers (stopped smoking within the previous 5 years), and current smokers, depending on their smoking history. Body mass index (BMI) (kg/m^2^) was evaluated by calculating the patient's weight divided by the square of the patient's height. Patients were categorized on their socioeconomic status (SES) based on a single patient's work experience, and economic and social positions and classified into three levels (high, middle, and low) (Sainz et al. 2021). The plaque index (PI) was evaluated using a scoring system (O'Leary, Drake, and Naylor 1972).

Prior to the patient's enrollment, a diagnostic histology biopsy at baseline verified the existence of OLP and supported the clinical diagnosis of OLP.

### 2.3 Study Outcomes

The primary outcome evaluated the impact of topical treatment performed with fluocinonide or placebo on OLP signs, symptoms, and severity scores. As a secondary outcome, possible baseline predictors of OLP outcome changes at 6 month follow‐up after treatment were evaluated, and whether baseline OLP clinical variables influenced the effectiveness of treatment protocols at 6 month follow‐up.

The effects of the treatment protocols on OLP were assessed through the analysis of OLP parameter score systems. OLP signs were scored, as previously reported (Polizzi et al. 2023), such as 0, absence of clinically detectable lesions; 1, white papular‐reticular lesions; 2, white plaque lesions; 3, reticular‐erosive lesions or desquamative gingivitis (erosion area < 1 cm^2^); 4, reticular‐erosive lesions or desquamative gingivitis (erosion area > 1 cm^2^); 5, reticular‐ulcerative lesions or bullous OLP (ulcer‐eroded areas < 1 cm^2^); 6, reticular‐ulcerative lesions or bullous OLP (ulcer‐eroded areas > 1 cm^2^). OLP symptoms were scored as 0, no symptoms; 1, mild (occasional symptoms); 2, moderate (e.g., while eating spicy food); 3, severe (i.e., while eating any food); 4, intolerable (always present) symptoms (Raj, Sreelatha, and Balan 2012). OLP sign and symptom scores were combined to determine the OLP severity score defined as 0, silent; 1–3, mild; 4–6, moderate; 7–9, severe; 10, very severe OLP. Finally, it was recorded the OLP extensions score by evaluating the number of sites with OLP located in each patient as (1) lips/labial vestibule, (2) buccal and lingual gums/alveolar mucosa, (3) tongue, (4) oral floor, (5) cheeks/vestibule, (6) hard palate, and (7) soft palate; then, it was calculated and derived a sum of OLP extension such as 0, no extension; 1, minimum extension; 2–3, moderate extension; 4–5, severe extension; 6–7, massive extension.

### 2.4 Sample Size Analysis, Reliability, and Randomization

The sample size was established on the different clinical OLP subtypes by considering an effect size of 0.30 with *α* = 0.050 and a power level of 0.80 for OLP severity score, set as the primary variable (Polizzi et al. 2023). It was determined that a minimum sample size of 17 patients per group would be required to achieve a good power sample. However, in order to avoid potential dropouts, we enrolled at least 23 patients per group, achieving a power level of 0.84.

All patients were evaluated by two independently blinded calibrated examiners (A.P., S.S.). The inter‐examiner reliability was performed on eight randomly selected patients per group. Using the intraclass correlation coefficient (ICC), the reliability analysis yielded a good agreement among examiners for OLP severity score (ICC = 0.814 [CI 0.802–0.828], first examiner; ICC = 0.818 [CI 0.804–0.826], second examiner).

Each patient was randomized in the study groups using sealed, numbered envelopes, and both medications were prepared and packed in sealed envelopes by a blinded clinician, independently considering clinical variables such as age, sex, PI, and presence of comorbidities which had no influence on the random allocation of each patient to the single treatment. A priori was determined to assign the letter “A” to the fluocinonide group and “B” to the placebo group. An operator not involved in the subsequent study's stages (A.A.) assigned the patients to each study group and generated a 1:1 random allocation sequence using a permuted block design through a computer generator.

### 2.5 Treatment

Participants allocated to the fluocinonide group received treatment with fluocinonide 0.05% gel, topically manually applied with a soft bristle toothbrush (about one teaspoon) to the OLP lesions twice a day for 8 weeks, in agreement with a previous study (Arduino et al. 2018). Patients allocated to the placebo group received the same treatment with a topical placebo containing an adhesive gel that contained 4% hydroxyethyl cellulose, similar in color and taste to the test group. Both treatments were prepared with the same galenic container furnished by the hospital pharmacy unit.

Before and after treatment, all patients performed a fungal swab in order to detect any concomitant fungal infections. Patients were instructed to maintain optimal oral hygiene and to avoid talking, eating, or drinking for 60 min after topical treatment to ensure prolonged contact with medication to the mucosa. After treatment, each patient was evaluated at 8 weeks and 3‐ and 6‐month follow‐ups. No mouthwashes, antibiotics, or other drugs were prescribed after treatment. At the end of each treatment session, patients were instructed and motivated to reinforce domiciliary oral hygiene using toothbrushes and interdental brushes. The same clinician warned patients about possible drug‐side effects (e.g., mucosal eruptions, fungal infections, altered taste, etc.), and they were asked to be referred to the clinics in case of any episodes during the 8 weeks of treatment. Moreover, at 8 weeks follow‐up, in order to supervise the correct use of treatment, each patient was asked to bring the drug (signed with “A” or “B”) used during the 8 weeks of therapy.

### 2.6 Statistical Analysis

Numerical clinical data were expressed as median and interquartile range (IQR), while categorical variables were expressed as numbers and percentages. Data were not normally distributed as verified by the Kolmogorov–Smirnov test; therefore, a non‐parametric approach was adopted. The comparisons between independent groups at each follow‐up session were performed using the Mann–Whitney test for numerical parameters and the Chi‐Square test for categorical variables. Four follow‐up sessions (baseline, 8 weeks, 3 months, 6 months) were compared using the Friedman test, for each group, OLP signs, symptoms, and severity and extension scores. Conditionally to the obtained significance, two‐by‐two comparisons between the four follow‐up sessions were conducted using the Dunn post hoc test. The Bonferroni correction was applied to adjust the *α*‐level of 0.050 by the number of possible comparisons (*n* = 6) among groups so that the adjusted significance level xwas set at < 0.008 (0.050/6). In order to assess any potential correlation between OLP indicators and the different clinical variables (age, gender, PI, BMI), comorbidities (diabetes, hypertension, hypercholesterolemia, hypothyroidism), anxiety, depression, and stress (average of the 3 DASS‐21 domains) in all enrolled patients, the Spearman correlation test was applied.

Following a logarithmic transformation of the primary variables (OLP signs, symptoms, and severity score) and obtaining the normality condition after transformation, a two‐way ANOVA was used to analyze the impact of both treatment protocols on OLP signs, OLP symptoms, OLP severity score, and OLP extension score (as continuous variables) changes and to estimate whether these variables changed based on two categorical variables such as treatment (I) and timing of treatment (II). It was evaluated how treatment protocols and timing, alone and in combination, influenced OLP signs, OLP symptoms, and OLP severity score changes in both groups by setting test protocol as a reference. Finally, uni‐ and multivariable linear regression models were established in order to estimate possible significant predictors of OLP signs, symptoms, severity score, and extension score changes (after logarithmic transformation) in the analyzed sample by potentially explicative variables such as age, gender, smoking, BMI, comorbidities, PI, stress, depression, and anxiety; smoking and comorbidities were inserted as dichotomous variables (yes/no). The statistical analyses were performed using statistical software (SPSS 22 for Windows package, Chicago, IL, USA). A *p*‐value < 0.05 was considered statistically significant.

## 3 Results

### 3.1 Study Participants

**FIGURE 1:**
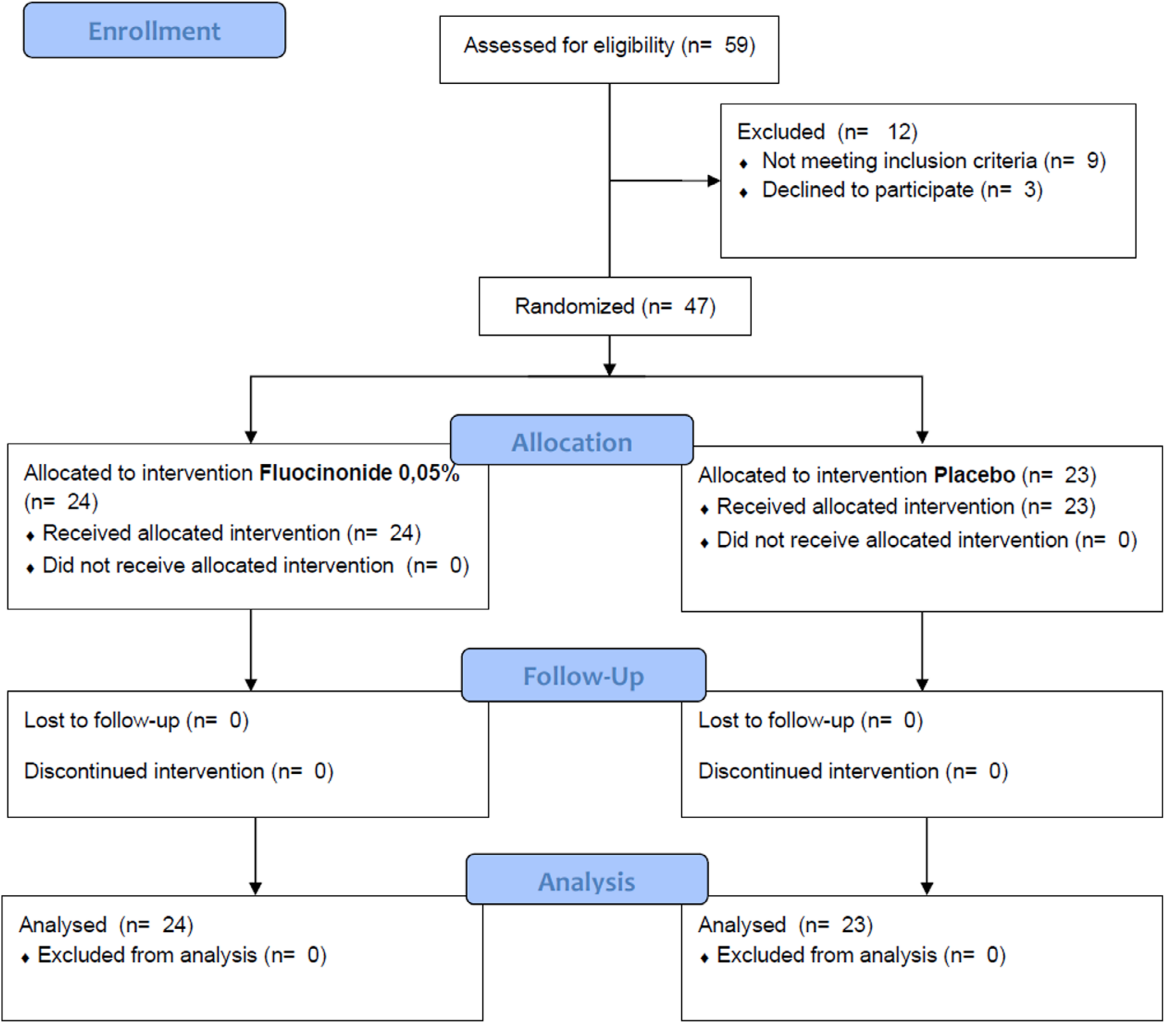
CONSORT flow diagram of the study.

At baseline, 59 subjects were first assessed for study eligibility (Figure 1). After screening, 12 individuals were excluded because they did not meet the inclusion criteria (*n* = 9) or declined to participate in the study (*n* = 3). Finally, 47 participants were enrolled and randomly allocated to receive fluocinonide (*n* = 24) or placebo (*n* = 23) topical treatment.

All patients completed successfully the 6 month follow‐up without any side effects. At baseline, both groups were similar in terms of age, gender, PI, and presence of comorbidities (Table 1), as well as OLP symptoms, signs, and OLP severity and extension scores (Table 2). Before and after treatment, no patient contracted fungal infections and no patients had any side effects regarding treatment.

**TABLE 1:** Baseline demographic and clinical variables of the sample. Values are represented by median and interquartile range (IQR) or number and percentage.

Variables	Placebo (*n* = 23)	Fluocinonide (*n* = 24)
Age, median (IQR)	61 (56–65)	59 (54–64)
Male gender, no. (%)	11 (47.8)	12 (50)
Plaque index, median (IQR)	1.4 (1–3)	1 (1–3)
Smoking		
Never smokers, no. (%)	14 (60.9)	13 (54.1)
Ex‐smokers, no. (%)	4 (17.4)	5 (20.9)
Current smokers, no. (%)	5 (21.7)	6 (25)
SES		
High, no. (%)	9 (39.1)	10 (41.7)
Middle, no. (%)	10 (43.5)	8 (33.3)
Low, no. (%)	4 (17.5)	6 (25)
BMI, median (IQR)	25.8 (23.5–26.9)	25.4 (22.9–26.3)
Diabetes, no. (%)	2 (8.7)	2 (8.3)
Hypertension, no. (%)	4 (17.4)	5 (20.8)
Hypercholesterolemia, no. (%)	3 (13.0)	4 (16.7)
Hypothyroidism, no. (%)	1 (4.3)	1 (4.2)
OLP forms, no. (%)		
Reticular	5	4
Plaque	7	8
Atrophic	6	7
Ulcerative	4	5
OLP desquamative gingivitis, no. (%)	12 (52.2)	14 (58.3)

Abbreviation: SES, socioeconomic status.

**TABLE 2:** Comparison of DASS‐21 scores between placebo and fluocinonide groups at baseline and at 6 months of treatment.

DASS‐21 Items, no. (%)	Placebo (*n* = 23)			Fluocinonide (*n* = 24)			Intergroup *p*	
	Baseline	6 months	*p*	Baseline	6 months	*p*	Baseline	6 months
Anxiety								
Normal	6 (26.1)	7 (30.4)	0.235	6 (25)	7 (29.2)	0.204	0.246	0.143
Mild	6 (26.1)	7 (30.4)	0.332	7 (29.2)	8 (33.3)	0.445	0.223	0.224
Moderate	5 (21.7)	5 (21.7)	0.278	4 (16.7)	5 (20.9)	0.657	0.567	0.542
Severe	4 (17.4)	3 (13.1)	0.665	5 (20.9)	3 (12.5)	0.325	0.587	0.332
Very severe	2 (8.7)	1 (4.4)	0.412	2 (8.3)	1 (4.2)	0.589	0.651	0.587
Stress								
Normal	5 (21.7)	6 (26.1)	0.342	6 (25)	7 (29.2)	0.065	0.325	0.348
Mild	6 (26.1)	7 (30.4)	0.109	7 (29.2)	8 (33.3)	0.235	0.114	0.467
Moderate	6 (26.1)	6 (26.1)	0.467	5 (20.9)	5 (20.9)	0.446	0.056	0.589
Severe	4 (17.4)	3 (13.1)	0.653	3 (12.5)	2 (8.3)	0.356	0.568	0.776
Very severe	2 (8.7)	1 (4.4)	0.448	2 (8.3)	1 (4.2)	0.217	0.345	0.436
Depression								
Normal	6 (26.1)	7 (30.4)	0.289	7 (29.2)	8 (33.3)	0.105	0.467	0.326
Mild	7 (30.4)	7 (30.4)	0.156	6 (25)	7 (29.2)	0.254	0.556	0.478
Moderate	6 (26.1)	6 (26.1)	0.412	7 (29.2)	7 (29.2)	0.678	0.476	0.489
Severe	3 (13.1)	2 (8.7)	0.113	3 (12.5)	1 (4.2)	0.101	0.234	0.525
Very severe	1 (4.4)	1 (4.4)	0.245	1 (4.2)	1 (4.2)	0.243	0.579	0.357

### 3.2 Primary Outcome

**FIGURE 2:**
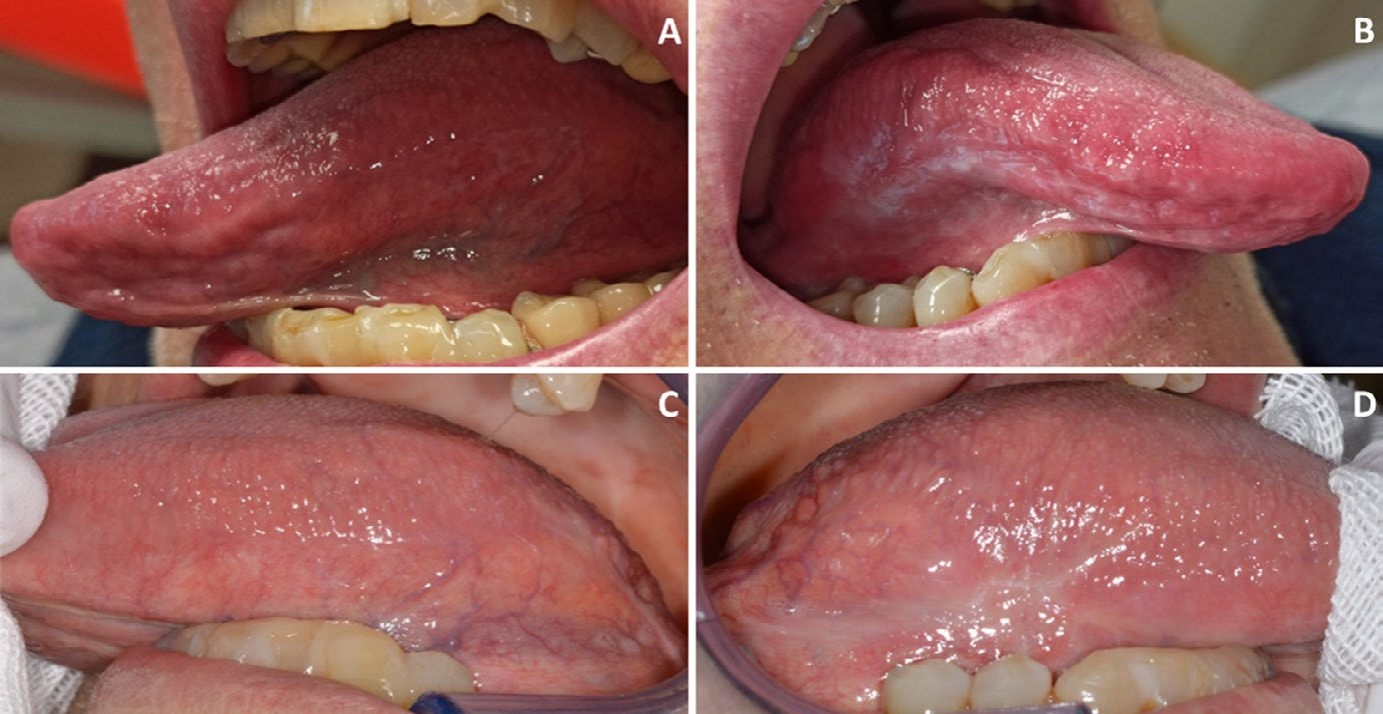
A 58‐year‐old patient with symptomatic OLP in the fluocinonide group. (A) Left and (B) Right tongue at baseline, (C) Left and (D) Right tongue at 6 months. The patient reported no oral pain or burning after treatment.

**FIGURE 3:**
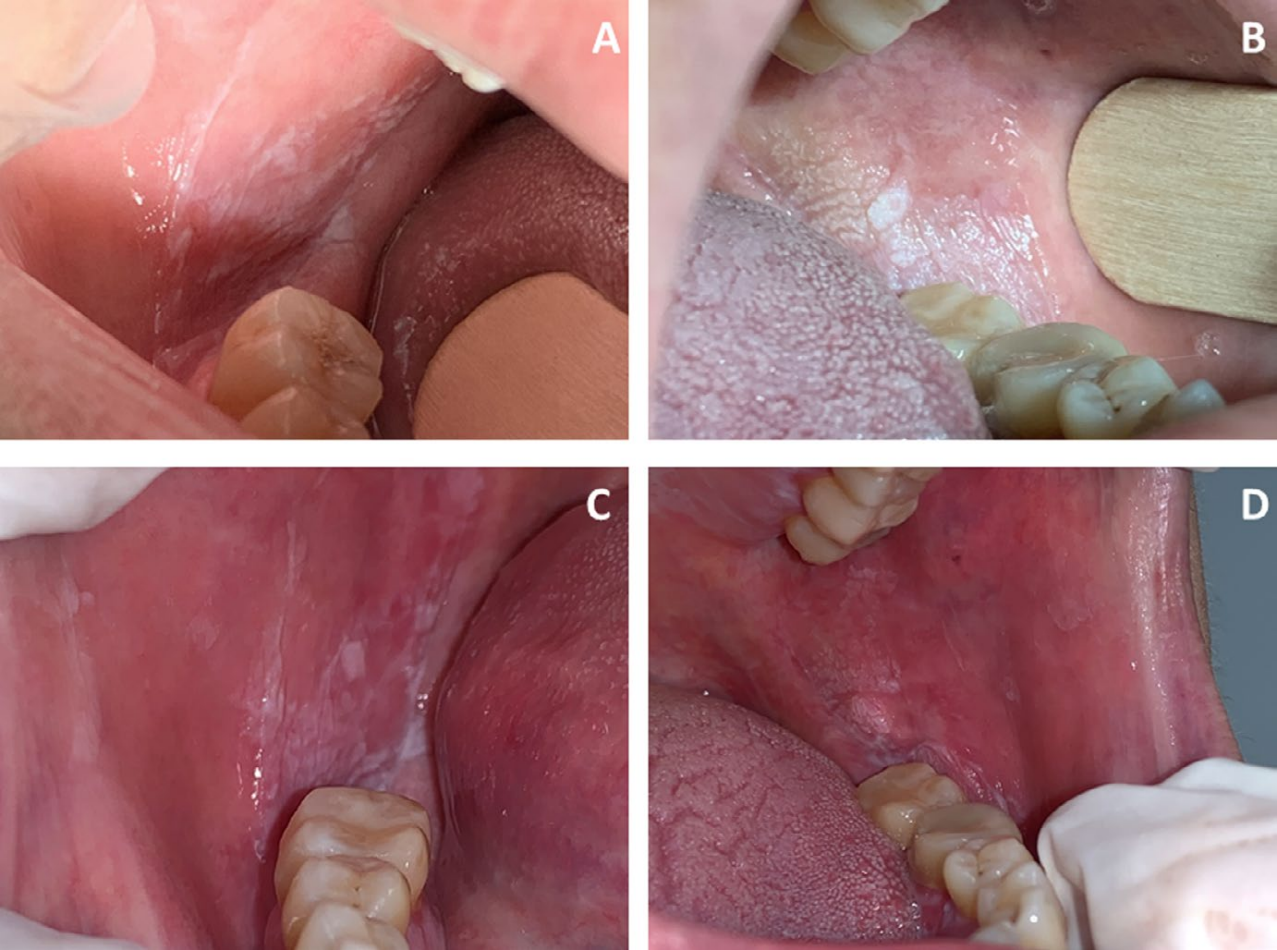
A 57‐year‐old patient with symptomatic OLP in the placebo group. (A) Right and (B) Left posterior buccal mucosa at baseline, (C) Right and (D) Left posterior buccal mucosa at 6 months. The patient reported a moderate worsening of oral burning after treatment.

Regarding intragroup comparison, in the fluocinonide group, there were statistically significant reductions in OLP symptoms (*p* = 0.011), signs (*p* = 0.045), and severity scores (*p* = 0.036), while there were no changes in the placebo group among the different follow‐up sessions (Table 3). After 8 weeks, in comparison with placebo, patients treated with fluocinonide showed a significant reduction in OLP signs (*p* < 0.001), symptoms (*p* < 0.001), and severity score (*p* = 0.006) (Table 2). However, at 6 months follow‐up, in comparison with placebo, patients in the fluocinonide group evidenced a significant reduction of OLP symptoms (*p* = 0.024), signs (*p* = 0.014), and OLP extension score (*p* = 0.028) (Table 2, Figures 2 and 3), while OLP severity score did not change between groups. There was no change in anxiety, depression, and stress among groups at baseline and at 6 months of treatment.

**TABLE 3:** OLP variable changes at baseline and at each follow‐up session using the Friedman Test and Dunn Post Hoc analysis. Values are represented such as median and interquartile range (IQR).

Time points	Placebo (*n* = 23)	Fluocinonide (*n* = 24)	*p*
OLP symptoms (range 0–4)			
Baseline	2.2 (1.2–3)	2.4 (1.3–3.4)	0.225
8 weeks	2 (1.3–3)	1.4 (0–1)^a^	< 0.001
3 months	2.7 (2.1–3)	2.1 (1.8–1.9)	0.115
6 months	2.4 (2.1–2.9)	1.8 (1.1–2.6)^c^, ^d^	0.014
*p*	0.059	0.011	
OLP signs (range 0–6)			
Baseline	4.2 (2.7–5)	4.3 (2.8–5)	0.365
8 weeks	3.6 (2.4–4.2)	1.4 (0.9–3.2)^a^	< 0.001
3 months	3.2 (2.3–3.5)	3 (2.8–3.3)^b^	0.059
6 months	4 (2.1–4.7)	3.1 (1.2–4.2)^c^, ^d^, ^e^	0.024
*p*	0.526	0.014	
OLP severity score (range 0–10)			
Baseline	6.1 (3.2–8.1)	6.2 (3.4–8.6)	NS
8 weeks	5.2 (3.2–7.4)	2 (1.2–4.3)^a^	0.006
3 months	4.6 (3.3–5.2)	3.4 (2.8)^b^	0.047
6 months	6.1 (4.3–8.3)	5.3 (4.4–6.2)^e^, ^f^	0.053
*p*	0.885	0.045	
OLP extension score (range 0–7)			
Baseline	3.2 (2.3–4.1)	3.2 (2.2–4.5)	NS
8 weeks	2.8 (2.1–3.8)	2.6 (1.8–2.9)	0.369
3 months	2.7 (2.3–3.4)	2.5 (2.1–2.9)^d^	0.552
6 months	3 (2.5–3.9)	3 (2–3)^e^, ^f^	0.028
*p*	0.096	0.036	

^a^ Significance between baseline and 8 weeks.

^b^ Significance between baseline and 3 months.

^c^ Significance between baseline and 6 months.

^d^ Significance between 8 weeks and 3 months.

^e^ Significance between 8 weeks and 6 months.

^f^ Significance between 3 months and 6 months.

### 3.3 Secondary Outcomes

The correlation analysis among OLP outcomes and clinical variables evidenced that, at 6 month follow‐up, there was a significant correlation among reduced OLP signs and hypertension (*r*s = 0.247; *p* = 0.048), hypothyroidism (*r*s = 0.109; *p* = 0.044), anxiety (*r*s = 0.359; *p* = 0.005), and stress (*r*s = 0.336; *p* = 0.012); among reduced OLP symptoms and smoking (*r*s = 0.257; *p* = 0.035), hypertension (*r*s = 0.187; *p* = 0.031), depression (*r*s = 0.241; *p* = 0.018) and stress (*r*s = 0.127; *p* = 0.027); among reduced OLP severity score, BMI (*r*s = 0.117; *p* = 0.014) and depression (*r*s = 0.373; *p* = 0.041); among reduced OLP extension score, BMI (*r*s = 0.204; *p* = 0.021) and stress (*r*s = 0.267; *p* = 0.017) (Table 4).

**TABLE 4:** Correlation analysis at 6‐month follow‐up among OLP scores, demographic, and clinical variables.

Variable	OLP signs		OLP symptoms		OLP severity score		OLP extension score	
	*R*s coeff.	*p*	*R*s coeff.	*p*	*R*s coeff.	*p*	*R*s coeff.	*p*
Age	0.258	0.058	0.114	0.331	0.225	0.441	0.225	0.225
Gender	0.104	0.345	−0.214	0.087	0.475	0.057	0.342	0.487
Smoking	0.425	0.068	0.257	0.035	0.334	0.017	0.119	0.025
Plaque index	0.331	0.052	0.066	0.254	0.158	0.315	0.247	0.066
SES	0.449	0.114	−0.114	0.068	0.247	0.586	0.331	0.105
BMI	0.189	0.108	−0.219	0.578	0.117	0.014	0.204	0.021
Diabetes	0.108	0.587	−0.366	0.147	0.457	0.108	0.225	0.688
Hypertension	0.247	0.048	0.187	0.031	−0.148	0.254	0.257	0.302
Hypercholesterolemia	0.215	0.087	0.224	0.114	0.441	0.227	0.104	0.207
Hypothyroidism	0.109	0.044	0.239	0.475	0.305	0.041	0.056	0.458
Anxiety	0.359	0.005	0.331	0.098	0.447	0.341	0.229	0.309
Depression	0.441	0.067	0.241	0.018	0.373	0.041	0.117	0.874
Stress	0.336	0.012	0.197	0.027	0.225	0.108	0.267	0.017

The estimation models using a two‐way ANOVA performed to determine the impact of fluocinonide on OLP signs, symptoms, severity score, and extension score changes at 6 month follow‐up revealed that treatment with fluocinonide determined a significant effect on the reduction of OLP signs (*p* = 0.017) OLP symptoms (*p* = 0.026), and OLP extension score (*p* = 0.028). The timing of treatment with fluocinonide significantly impacted OLP signs (*p* = 0.024), symptoms (*p* = 0.039), and extension score (*p* = 0.044) reduction at 6 months (Table S1).

The multivariate regression models, aimed at identifying the baseline impact of possible predictors of OLP signs, symptoms, severity score, and extension score changes at 6 months of treatment, highlighted that, in all patients, age was a significant predictor of OLP severity score (*p* = 0.025); smoking was a significant predictor of OLP signs (*p* = 0.025) and symptoms (*p* = 0.041); PI was a significant predictor of OLP symptoms (*p* = 0.023); diabetes was a significant predictor of OLP severity score (*p* = 0.026); hypertension was a significant predictor of OLP symptoms (*p* = 0.022); hypercholesterolemia was a significant predictor of OLP extension score (*p* = 0.015); hypothyroidism was a significant predictor of OLP signs (*p* = 0.026); anxiety was a significant predictor of OLP signs (*p* = 0.021) and OLP extension score (*p* = 0.041); depression was a significant predictor of OLP signs (*p* = 0.029), OLP symptoms (*p* = 0.002), and OLP extension score (*p* = 0.012); stress was a significant predictor of OLP signs (*p* = 0.036), OLP symptoms (*p* = 0.025), and OLP extension score (*p* = 0.006) (Table 5). Interestingly, the multivariate regression analysis also indicated that patients with high baseline anxiety, depression, and stress gained more benefits from fluocinonide at 6‐month follow‐up after treatment.

**TABLE 5:** Uni‐ and multivariable linear regression analysis for OLP score changes at 6‐month follow‐up in all enrolled patients. Significance was set as *p* < 0.05.

Variable	OLP signs				OLP symptoms			
	Univariate		Multivariate		Univariate		Multivariate	
	*B*	*p*	*B*	*p*	*B*	*p*	*B*	*p*
**Treatment (placebo)**								
Age	0.229	0.218	—	—	−0.225	0.558	—	—
Gender	0.345	0.336	—	—	−0.214	0.112	—	—
Smoking	−0.155	0.016	0.412	0.027	0.312	0.014	0.224	0.041
Plaque index	0.205	0.206	—	—	0.259	0.028	0.189	0.023
SES	−0.432	0.568	—	—	0.336	0.248	—	—
BMI	−0.371	0.257	—	—	0.458	0.369	—	—
Diabetes	0.484	0.158	—	—	−0.332	0.557	—	—
Hypertension	0.257	0.196	—	—	0.214	0.006	0.277	0.022
Hypercholesterolemia	0.332	0.419	—	—	−0.568	0.254	—	—
Hypothyroidism	0.257	0.045	0.257	0.026	0.254	0.274	—	—
Anxiety	0.236	0.036	0.189	0.021	0.214	0.041	0.135	0.028
Depression	0.115	0.029	0.158	0.029	0.339	0.032	0.457	0.002
Stress	0.336	0.036	0.254	0.036	0.247	0.041	0.336	0.025

Variable	OLP severity score				OLP extension score			
	Univariate		Multivariate		Univariate		Multivariate	
	*B*	*p*	*B*	*p*	*B*	*p*	*B*	*p*
**Treatment (placebo)**								
Treatment (placebo)	0.332	0.212	—	—	0.248	0.552	—	—
Age	−0.441	0.021	−0.324	0.025	0.665	0.652	—	—
Gender	−0.257	0.235	—	—	−0.336	0.287	—	—
Smoking	−0.665	0.258	—	—	0.236	0.547	—	—
Plaque index	0.289	0.369	—	—	0.158	0.274	—	—
SES	0.221	0.447	—	—	0.312	0.045	0.225	0.037
BMI	0.236	0.554	—	—	−0.336	0.778	—	—
Diabetes	−0.254	0.047	0.009	0.026	0.354	0.446	—	—
Hypertension	−0.336	0.554	—	—	−0.287	0.317	—	—
Hypercholesterolemia	0.218	0.332	—	—	0.554	0.025	0.258	0.015
Hypothyroidism	−0.331	0.053		—	0.215	0.886	—	—
Anxiety	−0.458	0.015	0.205	0.025	0.284	0.002	0.158	0.041
Depression	0.365	0.023	0.288	0.003	0.336	< 0.001	0.328	0.012
Stress	0.225	0.025	0.457	0.014	0.352	< 0.001	0.441	0.006

## 4 Discussion

The present RCT evaluated two treatment protocols in the topical management of OLP. The results showed that at 6 month follow‐up, compared to placebo, patients who were treated with fluocinonide experienced a significant improvement in OLP clinical outcomes. The analysis among OLP outcomes and clinical variables also evidenced that reduced OLP signs significantly correlated with psychological aspects such as stress, anxiety and depression at 6 month follow‐up.

The observed clinical efficacy of fluocinonide at 8 weeks, maintained up to 6 months of treatment aligns with existing literature on the use of topical corticosteroids for OLP (Buajeeb, Pobrurksa, and Kraivaphan 2000; Carbone et al. 2009, 2003; Chamani et al. 2015; Thongprasom et al. 2011). Some previous data in the literature reported that clobetasol propionate seemed to be effective in controlling pain and oral lesions compared to other topical corticosteroids, including fluocinonide (Carbone et al. 2009; Lozada‐Nur, Miranda, and Maliksi 1994). However, on the other hand, a recent Cochrane review (Lodi et al. 2020) aimed at assessing the topical management of OLP confirmed the role of topical corticosteroids (e.g., clobetasol propionate, fluocinonide, betamethasone, and triamcinolone acetonide) as a first‐line topical treatment due to its ability in pain management.

In this regard, the estimation of models aimed at assessing the impact of topical treatment protocols on OLP clinical outcomes revealed that treatment and the timing of treatment with fluocinonide determined, at 6‐month follow‐up, a significant effect on the reduction of OLP signs, OLP symptoms, and OLP extension score. In agreement, a previous study reported that fluocinonide, through its anti‐inflammatory and immunosuppressive properties, likely contributed to the reduction of OLP symptoms and lesion severity, offering relief to patients grappling with the burdensome impact of OLP on their oral health and overall well‐being (Thongprasom and Dhanuthai 2008). On the other hand, the actual level of quality of evidence for its use is moderate according to the American College of Physicians Guideline Grading System, whereas the level of evidence for clobetasol propionate is high (Davari, Hsiao, and Fazel 2014; Qaseem et al. 2010). OLP's chronic nature and potential for relapse have been well‐documented in the literature, often necessitating long‐term management strategies (Arduino et al. 2014; Kaliakatsou et al. 2002).

The multivariate regression models, aimed at identifying the baseline impact of possible predictors of OLP signs, symptoms, severity score, and extension score changes at 6 months of treatment, highlighted that, among others, anxiety, depression, and stress were significant predictors of OLP signs, symptoms, severity score and extension score. The analysis revealed also that patients with high baseline anxiety, depression and stress gained more benefits from fluocinonide at 6‐month follow‐up after treatment.

It is crucial to acknowledge that the pathogenesis of OLP involves intricate immune‐mediated processes, and the underlying factors contributing to its chronicity may not be entirely mitigated by short‐term interventions (Carrozzo et al. 2019; Carrozzo and Thorpe 2009; Liu et al. 2010; Mignogna et al. 2004). One plausible explanation for the observed impact of anxiety, depression, and stress could be due to the gradual impact of psychological aspects on the whole inflammatory and host defenses status of OLP patients with high stress, anxiety, and depression. In this regard, the sustained control of inflammation could be pivotal in preventing relapse in chronic conditions such as OLP, and the reduced host response is linked with a somatize impact of the psychological systemic status that may negatively impact the concomitant host response with a prolonged reduced anti‐inflammatory effect.

Not all patients may follow a uniform clinical course, and identifying factors associated with an increased risk of relapse could inform tailored treatment strategies. This may include factors such as the type and severity of lesions, patient‐specific immune responses, and potential triggers or exacerbating factors, especially in patients with high anxiety, depression, and stress (Manczyk et al. 2019).

The present study has some limitations that need to be addressed. One is linked with the follow‐up evaluation session. A longer follow‐up could have assessed the safety profile of fluocinonide in the long‐term run better. Topical corticosteroids and fluocinonide, even at lower concentrations, may pose risks such as mucosal atrophy, local immunosuppression, and the potential for systemic absorption. Monitoring for adverse effects over an extended duration is crucial to ensure that the benefits of fluocinonide therapy outweigh the potential risks. Furthermore, the observed relapse in this study in some patients at 8 weeks of treatment raises questions about the durability of the therapeutic effects of fluocinonide and the need for extended or maintenance treatment protocols. In this regard, future studies exploring extended treatment regimens or maintenance protocols may overcome insights into the optimal duration of fluocinonide therapy to sustain remission and prevent relapse.

## 5 Conclusions

The results of the present RCT evidenced that topical fluocinonide determined a significant reduction of OLP clinical outcomes in OLP patients at 6‐month follow‐up. The effects of topical fluocinonide up to 6 months following treatment of the present study support the promising anti‐inflammatory properties of fluocinonide in the treatment and evolution risk of OLP. Furthermore, the estimation models and the multivariate regression analysis also indicated that patients who showed high baseline anxiety, depression, and stress gained more benefits from fluocinonide at 6‐month follow‐up after treatment.

However, given the chronic and relapsing nature of OLP, future studies, maybe with a multicenter design, are requested to explore topical treatment regimens and maintenance protocols to further optimize the long‐term and risk‐stratified management strategies and outcomes of topical fluocinonide therapy in OLP patients.

## Author Contributions

**Alessandro Polizzi:** conceptualization, investigation, writing – original draft. **Gianluca Martino Tartaglia:** methodology, validation, visualization, writing – review and editing. **Simona Santonocito:** investigation, formal analysis, methodology. **Angela Alibrandi:** data curation, formal analysis, software. **Anna Elisa Verzì:** supervision, methodology. **Gaetano Isola:** conceptualization, writing – original draft, writing – review and editing, methodology, project administration.

## Conflicts of Interest

The authors declare no conflicts of interest.

## Supporting information


**Table S1.** Results of two‐way ANOVA for the dependent variable OLP signs, symptoms, severity score, and extension score at 6‐month follow‐up.

## Data Availability

The data that support the findings of this study are available from the corresponding author upon reasonable request.
