# Supplementary material for: Impact of Topical Fluocinonide on Oral Lichen Planus Evolution: Randomized Controlled Clinical Trial
Source: Oral Dis. 2024 Oct 14;31(2):510–21. doi: 10.1111/odi.15156 (PMC11976136; doi:10.1111/odi.15156)
Supplement: Supplementary file 1 — Table S1. Results of two‐way ANOVA for the dependent variable OLP signs, symptoms, severity score, and extension score at 6‐month follow‐up. [file ODI-31-510-s001.docx]

**Supplementary Table 1:** Results of two-way ANOVA for the dependent variable OLP signs symptoms, severity score and extension score at 6-months follow-up. For group, fluocinonide served as a reference. MS: Mean of Square. F: Fisher test; Group*Timing: Interaction term.

|  | **OLP signs** | | |
| --- | --- | --- | --- |
| *Source of variation* | *MS* | *F* | *p-value* |
| **Group** | 246.87 | 204.32 | 0.017 |
| **Timing** | 231.21 | 162.21 | 0.024 |
| **Group*Timing** | 4.55 | 5.28 | 0.036 |
| **Within** | 2.78 |  |  |
|  | **OLP symptoms** | | |
| *Source of variation* | *MS* | *F* | *p-value* |
| **Group** | 219.23 | 224.18 | 0.026 |
| **Timing** | 191.46 | 235.18 | 0.039 |
| **Group*Timing** | 3.68 | 3.36 | 0.058 |
| **Within** | 2.78 |  |  |
|  | **OLP severity score** | | |
| *Source of variation* | *MS* | *F* | *p-value* |
| **Group** | 208.11 | 212.11 | 0.116 |
| **Timing** | 195.14 | 257.18 | 0.547 |
| **Group*Timing** | 3.39 | 3.35 | 0.325 |
| **Within** | 2.47 |  |  |
|  | **OLP extension score** | | |
| *Source of variation* | *MS* | *F* | *p-value* |
| **Group** | 208.28 | 224.11 | 0.028 |
| **Timing** | 195.66 | 241.18 | 0.044 |
| **Group*Timing** | 3.47 | 3.28 | 0.236 |
| **Within** | 2.48 |  |  |
